# Thromboembolic Complications in Adult Patients Following Fontan Procedure—A Multicenter Study

**DOI:** 10.3390/jcm12103465

**Published:** 2023-05-14

**Authors:** Maciej Skubera, Aleksandra Gołąb, Tomasz Sternalski, Olga Trojnarska, Dariusz Plicner, Monika Smaś-Suska, Anna Mazurek-Kula, Agnieszka Bartczak-Rutkowska, Jacek Pająk, Piotr Podolec, Lidia Tomkiewicz-Pająk

**Affiliations:** 1 Department of Cardiac and Vascular Diseases, John Paul II Hospital, 31-202 Krakow, Poland; 2 Faculty of Medicine and Dentistry, Pomeranian Medical University in Szczecin, 70-204 Szczecin, Poland; 3 Center for Research and Innovative Technology, John Paul II Hospital, 31-202 Krakow, Poland; 4 Faculty of Medicine, Collegium Medicum Jagiellonian University, 31-008 Krakow, Poland; 5 1st Department of Cardiology, Poznan University of Medical Sciences, 61-701 Poznan, Poland; 6 Department of Cardiovascular Surgery and Transplantation, John Paul II Hospital, 31-202 Krakow, Poland; 7 Unit of Experimental Cardiology and Cardiac Surgery, Faculty of Medicine and Health Sciences, Andrzej Frycz Modrzewski Krakow University, 30-705 Krakow, Poland; 8 Department of Liver Diseases, John Paul II Hospital, 31-202 Krakow, Poland; 9 Department of Cardiology, Polish Mother’s Memorial Hospital, Research Institute, 93-338 Lodz, Poland; 10 Institute of Medical Sciences, Department of Surgery, Medical College of Rzeszow University, 35-025 Rzeszow, Poland; 11 Institute of Cardiology, Jagiellonian University Medical College, 31-008 Krakow, Poland

**Keywords:** Fontan procedure, thromboembolic complications, follow-up

## Abstract

Background: Morbidity and mortality following Fontan (FO) surgery are primarily thromboembolic in nature. However, follow-up data regarding thromboembolic complications (TECs) in adult patients after FO procedure are inconsistent. In this multicenter study, we investigated the incidence of TECs in FO patients. Methods: We studied 91 patients who underwent FO procedure. Clinical data, laboratory, and imaging investigations were collected prospectively during the scheduled medical appointments in 3 Adult Congenital Heart Disease Departments in Poland. TECs were recorded during a median follow-up of 31 months. Results: Four patients (4.4%) were lost to follow-up. The mean age of patients was 25.3 (±6.0) years at enrollment, and the mean time between FO operation and investigation was 22.1 (±5.1) years. A total of 21 out of 91 patients (23.1%) had a history of 24 TECs since an FO procedure, mainly pulmonary embolism (PE; *n* = 12, 13.2%), including 4 (33.3%) silent PE. The mean time since FO operation to the first TEC was 17.8 (±5.1) years. During follow-up, we documented 9 TECs in 7 (8.0%) patients, mainly PE (*n* = 5, 5.5%). Most patients with TEC had a left type of systemic ventricle (57.1%). Three patients (42.9%) were treated with aspirin, 3 (3.4%) with Vitamin K antagonists or novel oral anticoagulants, and 1 patient had no antithrombotic treatment at the time of TEC occurrence. Supraventricular tachyarrhythmias were present in 3 patients (42.9%). Conclusions: This prospective study shows that TECs are common in FO patients, and a significant number of these events occur during adolescence and young adulthood. We also indicated how much TECs are underestimated in the growing adult FO population. The complexity of the problem requires more studies, especially to standardize the prevention of TECs in the whole FO population.

## 1. Introduction

The Fontan (FO) operation is an end-stage palliative procedure used to treat univentricular physiology and has significantly improved the survival of patients [1,2]. However, FO patients have an increased risk of thromboembolic complications (TECs) compared to age-matched controls, and despite numerous prophylactic anticoagulation algorithms, TECs are a major cause of morbidity and mortality after FO procedures [3]. Several theories have been suggested to explain the hypercoagulable state in FO patients [4,5]. Well-known risk factors for the development of TEC include chronic venous congestion, high prevalence of supraventricular arrhythmias, the presence of prosthetic material, residual intracardiac shunt, or protein-losing enteropathy [6,7]. We have already shown that adult FO patients are also characterized by hepatic dysfunction as well as enhanced platelet activation, endothelial injury, and impaired fibrinolysis [8,9].

Follow-up data in patients after an FO procedure have shown an overall TECs rate ranging between 8 and 33% and account for about 8% of all FO patients’ deaths [4,10,11]. Incidence varies and can be attributed to different definitions and also distinct diagnostic methods of the TECs as well as insufficient follow-up data [12,13]. The aim of this study was to assess the prevalence rate of TECs in FO patients and to investigate the associations between demographic, anatomical, and clinical factors along with TECs in this group of patients.

## 2. Materials and Methods

### 2.1. Patients

In this multicenter report, we studied 91 patients who underwent FO procedure. Clinical, laboratory, and imaging data were collected prospectively during the scheduled medical appointments in 3 Adult Congenital Heart Disease Departments in Poland (Krakow, Poznan, and Lodz) between June 2018 and September 2019. Follow-up was scheduled according to the regular outpatient visits with a 1 year time interval.

The previous and current history of antithrombotic therapy were recorded. Comorbidities were assessed in groups based on the affected system with separate distinctions of thromboembolism and supraventricular tachyarrhythmias (SVTs). The exclusion criteria for FO patients were as follows: any acute illness, diabetes mellitus, serum creatinine level > 120 µmol/L, malignant neoplasms, alcohol abuse, or pregnancy.

TECs were classified as pulmonary embolism (PE), stroke or transient ischemic attack (TIA), Fontan circuit thrombus (FCT), deep vein thrombosis (DVT), or peripheral systemic thrombosis (PST). The diagnosis of PE was based on the presence of typical symptoms and positive results of ventilation/perfusion scintigraphy, according to current guidelines [14]. This method was preferred over high-resolution computed tomography to reduce whole-life irradiation dose. Stroke and TIA were defined according to the American Heart Association/American Stroke Association Guidelines [15]. FCT was defined as the presence of the thrombotic material in the proximal inferior vena cava, FO conduit, tunnel, or right atrium in subjects operated on using the classic atriopulmonary technique [16]. Diagnosis of DVT and PST were established in symptomatic subjects with positive ultrasound results, according to the respective guidelines [14,17]. The CHA_2_DS_2_-VASc score was used to estimate the risk of initial stroke in patients with atrial fibrillation [18].

Events were diagnosed based on individual patient reports or supplementary imaging when clinical symptoms or alterations in laboratory results were observed. Confirmatory imaging studies were obtained and analyzed before TEC occurrence. Depending on the type of TEC, the diagnosis was established using echocardiography, vascular ultrasound, angiography, or ventilation-perfusion scintigraphy. SVTs were described as either standard or Holter monitoring ECG findings of atrial fibrillation or flutter. The decision on the type of antithrombotic agent was left to the cardiologist providing regular treatment.

This study was performed in accordance with the Declaration of Helsinki and was approved by the Research Ethics Committee (130/KBL/OIL/2018). Patients provided written informed consent. Personally identifiable information of the participants was anonymized upon extraction of the relevant data for the study.

### 2.2. Laboratory Investigation

Fasting blood samples were collected into 0.1 volume of 3.2% trisodium citrate from the antecubital vein with minimal stasis on the same day that clinical data were recorded. In anticoagulated patients, blood was drawn at least 5 days after anticoagulation withdrawal. Citrated blood samples were centrifuged at 3000 g for 20 min and stored in aliquots at −80 °C until further use. Red blood cell count, white blood cell count, platelet count, hematocrit level, total protein level, alanine aminotransferase level, creatinine level, C-reactive protein level, and international normalized ratio were assayed by routine laboratory techniques.

Fibrinogen was determined using the thrombin clotting time method. Coagulation factors (F) V, FVII, FVIII, FIX, and FX were measured by 1-stage clotting assays using factor-deficient plasmas (Siemens, Marburg, Germany). Antithrombin activity was measured using Berichrom (Siemens). Protein C activity was measured using a chromogenic substrate assay (Siemens). Protein S was determined using a latex ligand immunoassay (Instrumentation Laboratory, Milan, Italy). The immunoenzymatic assay was used to determine plasma prothrombin fragments 1.2 (F1.2; Siemens). Plasma PAI-1 antigen levels were measured by an ELISA (American Diagnostica, Greenwich, Conn). Thrombin activatable fibrinolysis inhibitor (TAFI) antigen was determined with an ELISA (Chromogenix, Lexington, Mass). Plasma TAFI activity was measured by a chromogenic assay using the Actichrome Plasma TAFI Activity Kit (American Diagnostica). Von Willebrand Factor (vWF) activity and antigen (Diagnostica Stago) were also measured in plasma.

All the hemostatic measurements were performed by technicians blinded to the sample status. The coefficients of intra-assay and interassay variations were less than 9%.

### 2.3. Echocardiography and Shear Wave Elastography

The ejection fraction of the single ventricle was assessed using a semi-qualitative assessment. Valvular competence was also evaluated in the patients by two experienced, independent cardiologists using echocardiography (Vivid 7, GE Medical Systems, Milwaukee, WI, USA) as previously described [19].

Shear wave elastography was performed using a Philips iU22 XMatrix Ultrasound System (Amsterdam, Netherlands) by an experienced physician. For each patient, 10 measurements were taken for the liver stiffness estimation.

### 2.4. Statistical Analysis

Descriptive statistics were used to outline the baseline characteristics. Categorical variables are presented as numbers and percentages. Continuous variables are expressed as mean ± standard deviation (SD) or median and interquartile range (IQR). Normality was assessed by Shapiro–Wilk test. Categorical variables were analyzed using the χ^2^ test or Fisher’s exact test as appropriate. Differences between groups were compared using the Student’s or Welch’s *t*-test depending on the equality of variances for normally distributed variables. The Mann–Whitney *U*-test was used for non-normally distributed continuous variables. Kaplan–Meier survival curves were created to determine event-free survival from TECs. Statistical analyses were performed using Statistica^®^, Version 13.3.721 (TIBCO Software Inc., Palo Alto, CA, USA).

## 3. Results

### 3.1. Baseline Characteristics of Fontan Patients

As shown in Table 1, we studied 91 FO patients who underwent FO procedures at a mean age of 5.2 (±3.9) years. The mean time between operation and investigation was 22.1 (±5.1) years. More than half of FO patients had a pre-operative ventricular septal defect in single ventricle physiology (56%). The majority of them were operated using the lateral tunnel technique (89.0%), and nearly half of them had fenestration performed (45.1%). Systemic ventricle anatomy was left in major (66.3%) of patients. The most frequently recorded complications were hepatic ones (37.7%), and aspirin therapy was implemented in half of the studied patients (53.8%). Thirty (33.0%) of the studied patients were treated with oral anticoagulants (Table 1).

The mean age of FO patients at enrollment was 25.3 (±6.0) years. Patients who experienced TEC had lower baseline oxygen saturation and more commonly had varices compared to patients free of this complication. Moreover, laboratory investigation showed lower values of coagulation factors: V, VII, IX, X, and protein S, as well as thrombin activatable fibrinolysis inhibitor in this group of patients. We also observed increased activity of the von Willebrand Factor in patients with a history of TEC. There were no other differences in baseline clinical and laboratory variables (Table 2).

### 3.2. Thromboembolic Events Profile at Enrollment

Twenty-one patients (23.1%) had developed 24 TECs since their FO procedure (1.2 per 100 patient-years). As shown in Table 3, the mainly observed TEC type was PE (*n* = 12, 13.2%), including 4 silent ones (33.3%). Other thrombus locations included FO circuit in 6 (6.6%) cases, intracranial in 4 (4.4%), and peripheral vasculature in 2 patients (2.2%).

Table 3 shows that among this subgroup of studied patients, 71.4% had a left type of systemic ventricle, and 57.1% had fenestration, mainly in those patients who underwent stroke/TIA (in 100% and 75% cases, respectively) and DVT (in 100% and 100% cases, respectively) or PST (in 100% and 100% cases, respectively). The prevalence of SVTs was 37.5%. One patient with DVT and 1 with a PST history had a left type of systemic ventricle and fenestration performed, as well as diagnosed SVTs. The occurrence of a specific type of TEC event was not related to sex in the studied group.

**Figure 1:**
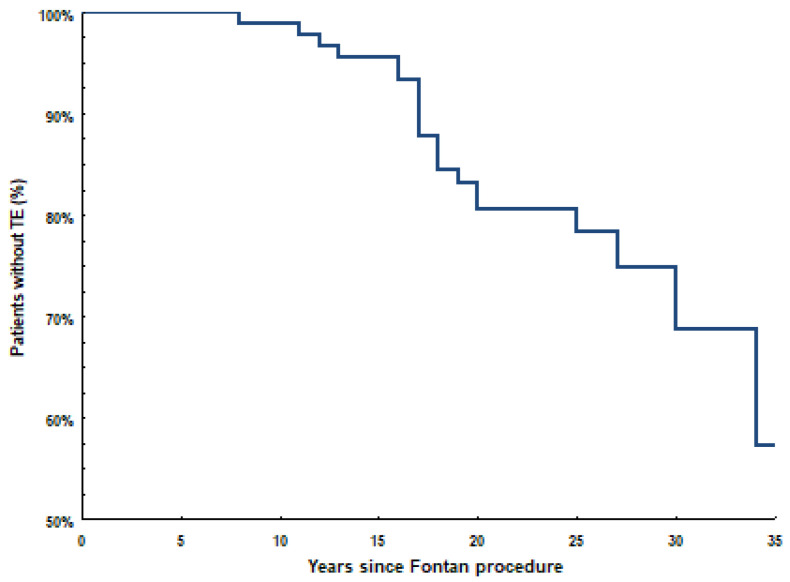
Kaplan–Meier curve for survival from the thromboembolic event.

The Kaplan–Meier curve shows overall freedom from TEC since the FO procedure (Figure 1). The mean time since FO operation to the first TEC was 17.8 (±5.1) years. An increase in TECs incidence can be observed approximately 16.0–17.0 years after FO operation.

### 3.3. Follow-Up

Four patients (4.4%) were lost to follow-up. One patient (1.1%) died due to respiratory tract bleeding complications during the Bentall De Bono procedure, and 1 subject (1.1%) as a result of massive gastrointestinal bleeding in a clinical progression of what is widely described as the ‘Failing Fontan’ phenotype. Additionally, 2 female patients (2.2%) were excluded due to pregnancy.

**Figure 2:**
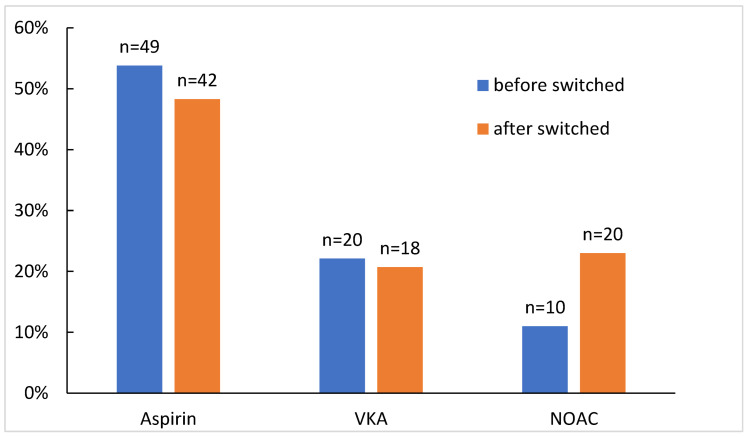
Modification of thrombophylaxis in this study group during follow-up. NOAC: novel oral anticoagulants; VKA: vitamin K antagonist.

Fifteen patients (16.5%) had switched antithrombotic medication during the follow-up period, with a mean time to medication switch 15.8 (±9.3) months. In 11 cases, antithrombotic therapy was switched to novel oral anticoagulants (NOAC) [7 from aspirin and 4 from vitamin K antagonist (VKA)] and in 2 cases to VKA (1 from aspirin and 1 from NOAC). There was 1 (1.1%) case of a therapy-naive patient who had secondary NOAC prophylaxis started after the development of DVT with FCT. One patient had primary prophylaxis after aspirin was initiated (Figure 2).

During a mean follow-up of 30.3 months (±5.6), we documented TEC in 7 (8.0%) patients (4.1 per 100 patient-years). As shown in Table 4, we recorded 5 (5.5%) PEs, 1 (1.1%) FCT, and 3 (3.3%) cases of DVT. There was no cerebrovascular event recorded during the follow-up. A thrombus in the right side of the heart was not detected by echocardiography. In this group of patients, the mean time from FO operation to TEC was 22.1 (±11.8) years. TECs were recorded slightly more often in men (57.1%). The most common TEC was PE (71.4%), out of which 1 was recurrent embolism (Table 3). In this group, 3 patients (42.9%) had a history of previous TEC. Adverse outcomes occurred in 14.3% of patients with a history of previous TEC and in 5.7% of patients with no events at baseline. Most patients with TEC had a left type of systemic ventricle (57.1%).

At the time of TEC occurrence, 3 (42.9%) patients were on anticoagulant therapy (1 patient on VKA and 2 patients on NOAC treatment), while the remaining 3 (42.9%) patients were treated with aspirin. After TEC, the anticoagulant treatment was continued in those 3 cases, whereas 3 patients were switched from aspirin to NOAC. One (14.3%) therapy-naive patient was started on NOAC due to TEC occurence.

The CHA_2_DS_2_-VASc score was ≥ 2 in 3 patients (42.9%), whereas others had a CHA_2_DS_2_-VASc score equal to 0. SVTs were present in 3 patients (42.9%).

## 4. Discussion

This study describes the demographic, anatomical, and clinical characteristics of FO adult patients, as well as their antithrombotic treatment along with TECs profile, for 22 years after FO surgery. We confirmed that TECs are common in FO patients, which may adversely affect the prognosis after FO palliation. Further studies are needed to evaluate the causes of the prothrombotic state in FO adults on the basis that it will be possible to extract a subgroup of high-risk patients and standardize antithrombotic prophylaxis to reduce the TECs rate.

It has been previously established that changes in circulation among patients with FO physiology, i.e., non-pulsative flow in the pulmonary circulation, slow venous flow, and hypoxemia, lead to endothelial as well liver injury, two primary sites for synthesis of pro- and anticoagulant agents and finally results in the prothrombotic state in the FO population [9,20]. Of note, these alterations simultaneously predispose to bleeds; however, those events are recorded less frequently than TECs in FO patients [8,9]. Deshaies et al. revealed the greatest increase in TECs post-surgery between 15 and 20 years of age [21]. At the same time, Khairy et al. found that the cumulative hazard for thromboembolic death increased steadily for 15 years after FO surgery [3]. We confirmed these findings, and we also showed that the constant progression of circulatory alterations is manifested by the first episode of TEC, mostly in adolescents and young adults. In contrast to our study, Idorn et al. and Rosenthal et al. showed that the mean time from FO surgery to the first TEC was shorter [17.8 (±5.1) vs. 5.0 (±5.5) and 6.1 (±5.0) years, respectively] [22,23]. Several factors, such as the size of the study group, age at FO operation, type of FO surgery, type of thromboprophylaxis, or other documented risk factors such as low post-operative cardiac index, may have an influence on the differences in time to the appearance of the first adverse episode [24]. Only 7.7% of studied patients were in the NYHA functional class III or IV at enrollment; thus, there were not many subjects who had developed severe heart failure, which may partly explain this relationship.

TECs are frequently recorded adverse events in patients with FO physiology [6,9]. In addition, Yang et al. revealed that among the whole group of adults with congenital heart disease, 50% of TECs occurred in FO patients, who constituted only 14% of the study population [25]. Therefore, primary and secondary prophylaxis were used in daily practice, although controversy exists about the optimal prevention strategy. Classically, the basis of antiplatelet or anticoagulant therapy is carried out with aspirin or VKA [24]. Recent reports showed that aspirin is generally not an inferior antithrombotic therapy to VKA (in our study, 53.8% vs. 22.1% used at baseline) [11,21]. Nevertheless, due to important limitations, i.e., high rate of aspirin resistance and difficulties in achieving a consistent international normalized ratio range with VKA among the FO population, the newest research tries to improve thromboprophylaxis through NOAC implementation, which provides easier use, e.g., no need of laboratory monitoring of its effectiveness and lower number of drug interactions [12,26]. The lack of knowledge about NOAC safety and effectiveness in recent guidelines might be the reason why the population taking NOAC at baseline was approximately two times lower than the one taking VKA in this study [27,28]. Eleven percent of FO adults had been taking NOAC at baseline, and an additional 12.0% had NOAC introduced, as an alternative to aspirin or VKA, during follow-up. Finally, TECs occurred with a similar percentage among NOAC and aspirin groups (10.0% vs. 7.3%). Less often, adverse events occurred in the group of patients on VKA (5.6%). Notably, our study cannot settle uncertainties that regard the safety and efficacy of NOAC treatment in FO patients due to the small size of the study group and the disproportionate distribution of thromboprophylaxis, which caused the data to be underestimated. This issue needs to be investigated in a larger study group.

Data about typical thrombus localization are also inconsistent. There are studies that mainly recorded venous localizations, while others showed mostly systemic thrombi, defined as cardiac, neurological, peripheral arterial, renal, or mesenteric thromboembolism [21,22,24]. Quite frequent are intracardiac thrombi, mainly in the right atrium [6]. In our study, we showed that thrombi were located primarily in pulmonary circulation in the form of PE events, of which almost 34% were silent PE, i.e., without clinically visible complications. Almost two times fewer silent PE events were documented by Varma et al., but in a shorter time from operation [29]. The mentioned study revealed that there is a one in six chance of a silent PE being present in asymptomatic adults FO subjects. Our report, along with this evidence, showed how much TECs are underestimated among the FO population. Hemodynamic and clinical long-term consequences of asymptomatic PE in this population are still unknown. It is certain that increased pulmonary resistance, which is associated with silent PE, adversely affects pulmonary circulation and, finally, might lead to pulmonary vascular disease [29,30]. In addition to the hemodynamic alternations associated with FO circulation affecting endothelial cells, it is suggested that in PE patients, the endothelial injury may be exacerbated by increased sheer stress on the wall of pulmonary vasculature [29,31]. In this current study, we observed that patients, who had a history of TEC at the time of recruitment, had significantly lower resting saturation and enhanced endothelial activity, reflected by increased von Willebrand Factor activity. We previously thoroughly described the relation between endothelial injury and impaired fibrinolysis, which may be a relevant cause of TECs occurrence among the FO population [8,9].

Additional increases in TEC risk occur in the presence of atrial arrhythmia, which occurs in more than 50% of FO adults 20 years after FO palliation [32]. Despite this fact, we failed to show this relation. Moreover, Egbe et al. showed that most cases during TEC diagnosis were asymptomatic in FO patients with atrial arrhythmias [32]. Therefore, there is a need to search for high-risk TEC patients in the FO population actively. The CHA_2_DS_2_-VASc score, as a simple and easy-to-use tool, is used in daily practice to establish TEC risk and, eventually, the implementation of therapy in patients with atrial fibrillation diagnosis; however, its usefulness is unclear in the adult congenital heart disease population [33]. Of note, FO subjects are younger compared to the typical population of patients with atrial fibrillation and have a higher TEC risk due to FO circulation. There is still a lack of reports exploring this issue among adult congenital heart disease patients, and this study also shows that there is no correlation between TEC and SVT. The classic CHA_2_DS_2_-VASc seems to have an insufficient score in this population; however, this issue requires further analysis. Moreover, Jensen et al. [34] recommended introducing anticoagulation therapy in FO patients even if the CHA_2_DS_2_-VASc score is 0. Taken together, a significant thromboembolic risk increase in the FO population with SVT and uncertain usability of the mentioned score makes a need to create appropriate and effective risk estimation in this specific population.

Our study has several limitations. First of all, the size of the studied group was limited, although representative of adult FO patients. Moreover, patients were heterogeneous with respect to cardiac diagnosis and the type of FO surgery, as well as antithrombotic treatment. A higher number of patients would be necessary to draw a firm conclusion.

## 5. Conclusions

We conclude that TECs are common in FO-operated patients, and a significant number of TECs occur during adolescence and young adulthood. The current study also showed how much TECs are underestimated in the growing adult FO population. The complexity of the problem requires more studies, especially to standardize the prevention of TECs in the whole FO population, which may improve prognosis after FO surgery.

## Figures and Tables

**Table 1:** Demographics and clinical characteristics of Fontan patients at enrolment.

Variable	Fontan Patients (*n* = 91)
Age at Fontan operation	5.2 ± 3.9
Time between Fontan operation and enrollment	22.1 ± 5.1
Pre-operative anatomy, *n* (%)	
VSD in single ventricle physiology	51 (56.0)
Transposition of great arteries	40 (44.0)
Hypoplasia of the right ventricle	24 (26.4)
Tricuspid atresia	22 (24.2)
Double-outlet right ventricle	13 (14.3)
Hypoplastic left heart syndrome	13 (14.3)
Systemic ventricle type, *n* (%)	
Left	59 (66.3)
Right	28 (31.5)
Unidentified	2 (2.3)
Type of Fontan, *n* (%)	
Lateral tunnel	81 (89.0)
Extracardiac conduct	8 (8.8)
Atrio-pulmonary connection	2 (2.2)
Fenestration, *n* (%)	41 (45.1)
NYHA functional class, *n* (%)	
I	19 (20.9)
II	65 (71.4)
III	7 (7.7)
IV	0 (0)
Medication, *n* (%)	
Aspirin	49 (53.8)
MRA	39 (42.9)
Beta blockers	26 (28.6)
ACEI/ARB	17 (18.7)
VKA	20 (22.1)
Loop diuretics	13 (14.3)
NOAC	10 (11.0)
None	12 (13.2)
Comorbidities, *n* (%)	
Hepatic	34 (37.4)
Thromboembolic	24 (26.4)
Thyroid	19 (20.9)

Values are displayed as mean ± standard deviation or number (percentage). ACEI/ARB: angiotensin-converting enzyme inhibitor/angiotensin receptor blocker; MRA: aldosterone receptor antagonists; NOAC: novel oral anticoagulants; NYHA: New York Heart Association; VKA: vitamin K antagonist; VSD: ventricular septal defect.

**Table 2:** Clinical and laboratory characteristics of Fontan patients in relation to thromboembolic complications.

Variable	Fontan Patients (*n* = 91)	Thromboembolic Complication		*p*-Value
		No = 70	Yes = 21	
Age at enrollment (years)	24.7 ± 6.1	24.4 ± 6.1	25.5 ± 6.3	0.464
Male, *n* (%)	45 (49.5)	35 (50.7)	10 (47.6)	0.667
BMI (kg/m^2^)	22.6 ± 3.2	22.7 ± 3.3	22.3 ± 3.2	0.579
SaO_2_ (%)	91.7 ± 5.0	92.6 ± 4.1	89.2 ± 6.5	0.005
Echocardiography				
SVEF (%)	55.0 [50.0–60.0]	55.0 [50.0–60.0]	50.0 [45.0–55.0]	0.077
Visible fenestration, *n* (%)	32 (35.2)	22 (31.4)	10 (47.6)	0.246
Atrioventricular valve regurgitation, *n* (%)				
mild	41 (45.1)	32 (45.7)	9 (42.9)	0.654
moderate	27 (29.7)	21 (30.0)	6 (28.6)	0.777
severe	2 (2.2)	2 (2.9)	0 (0)	0.419
Elastography				
Liver stiffens (kPa)	9.2 [7.8–10.3]	9.2 [7.9–10.3]	8.9 [7.8–9.9]	0.841
F1, *n* (%)	1 (1.1)	1 (1.4)	0 (0)	0.570
F2, *n* (%)	30 (33.0)	22 (31.4)	8 (38.1)	0.697
F3, *n* (%)	10 (11.0)	7 (10.0)	3 (14.3)	0.648
F4, *n* (%)	7 (7.7)	5 (7.1)	2 (9.5)	0.777
VAST score (point)	1.0 [0.0–2.0]	1.0 [0.0–2.0]	1.0 [0.0–2.0]	0.782
Varices, *n* (%)	18 (19.8)	9 (12.9)	9 (42.9)	0.004
Ascites, *n* (%)	6 (6.6)	4 (5.7)	2 (9.5)	0.151
Splenomegaly, *n* (%)	40 (44.0)	33 (47.1)	7 (33.3)	0.188
Thrombocytopenia, <150 × 10^3^/mm^3^, *n* (%)	39 (42.9)	33 (47.1)	6 (28.6)	0.090
Laboratory investigation				
RBC (10^6^/mm^3^)	5.3 ± 0.6	5.3 ± 0.5	5.5 ± 0.8	0.054
HBG (g/dL)	14.6 ± 3.3	14.3 ± 3.3	15.5 ± 3.1	0.131
WBC (10^3^/mm^3^)	5.9 ± 2.1	6.0 ± 2.2	5.5 ± 1.8	0.335
PLT (10^3^/mm^3^)	161.0 [124.5–203.5]	151.0 [123.0–203.0]	177.5 [149.5–210.3]	0.319
TC (μmol/L)	3.7 ± 0.8	3.7 ± 0.8	3.5 ± 0.7	0.276
TG (μmol/L)	0.9 ± 0.4	0.8 ± 0.4	1.0 ± 0.4	0.127
LDL cholesterol (μmol/L)	2.3 ± 0.7	2.3 ± 0.7	2.1 ± 0.7	0.250
CRP (mg/L)	1.0 [0.7–2.3]	1.4 [0.7–2.3]	1.1 [0.8–2.2]	0.471
Creatinine (μmol/L)	72.0 [64.0–82.0]	72.0 [64.0–82.0]	72.0 [65.5–82.3]	0.831
ALT (U/L)	25.0 [19.5–32.0]	27.0 [21.0–32.0]	20.0 [18.3–25.8]	0.098
AST (U/L)	25.0 [22.0–29.5]	27.0 [22.0–30.0]	23.5 [20.3–28.3]	0.117
Albumin (g/L)	42.5 [40.4–44.5]	42.6 [40.4–44.7]	42.3 [39.9–43.2]	0.847
Total bilirubin (μmol/L)	16.1 [11.0–24.0]	16.5 [10.8–24.4]	15.8 [14.4–20.5]	0.634
GGTP (U/L)	59.0 [39.0–115.0]	59.0 [39.5–115.0]	45.0 [40.8–68.3]	0.587
aFP (ng/mL)	3.3 ± 2.3	3.2 ± 2.1	2.1 [1.8–3.3]	0.727
APRI	0.5 ± 0.3	0.5 ± 0.3	0.4 ± 0.1	0.281
INR	1.4 ± 0.3	1.4 ± 0.3	1.5 ± 0.2	0.105
PT (s)	16.1 ± 2.8	15.7 ± 3.0	17.0 ± 2.1	0.103
D-dimer (μg/L)	246.0 [170.0–398.0]	247.0 [170.0–361.0]	173.0 [170.0–421.8]	0.895
Endothelin (pg/mL)	2.5 ± 1.3	2.4 ± 0.7	3.0 ± 2.4	0.088
Liver-derived hemostatic factors				
FV (%)	57.0 [41.3–74.8]	62.0 [42.5–76.5]	48.0 [41.5–55.5]	0.027
FVII (%)	58.0 [49.3–69.0]	58 [50.0–72.0]	55.0 [42.5–65.0]	0.022
FVIII (%)	89.0 [67.3–108.8]	87.0 [67.5–109.0]	94.0 [66.0–107.5]	0.944
FIX (%)	87.0 [70.0–107.5]	91.0 [78.0–111.0]	69.0 [58.0–96.0]	0.007
FX (%)	80.0 [64.8–91.0]	83.0 [73.0–93.0]	59.0 [50.0–77.5]	<0.001
Fibrinogen (g/dL)	2.4 ± 0.5	2.4 ± 0.6	2.5 ± 0.5	0.370
Antithrombin (%)	94.0 [88.0–100.0]	94.5 [91.0–100.0]	87.5 [83.5–92.8]	0.059
Protein S (%)	87.0 [74.3–96.0]	90.0 [80.0–99.0]	69.0 [60.0–87.0]	0.059
Protein C (%)	96.0 [82.3–109.5]	96.0 [85.5–111.5]	80.0 [70.0–103.5]	0.007
TAFI activity (%)	93.0 [81.2–101.6]	94.7 [84.6–101.7]	83.7 [75.9–91.6]	0.011
TAFI antigen (%)	85.0 [72.6–99.0]	86.6 [75.6–100.5]	78.1 [65.9–92.9]	0.281
Endothelium-derived hemostatic factors				
tPA (ng/mL)	6.0 [4.1–7.4]	5.5 [3.9–7.2]	6.6 [4.6–9.3]	0.256
PAI-1 (ng/mL)	10.0 [7.4–15.2]	9.9 [7.2–14.5]	12.5 [8.6–16.6]	0.868
vWF activity (%)	98.0 [85.3–125.6]	95.8 [82.9–125.4]	114.1 [94.8–135.5]	0.029
vWF antigen (%)	140.0 [120.3–170.7]	138.0 [119.1–166.5]	144.4 [133.1–192.1]	0.123

Values are displayed as mean ± standard deviation or number (percentage). aFP: alpha fetoprotein; ALT: alanine aminotransferase; APRI: aspartate aminotransferase to platelet ratio index; AST: aspartate aminotransferase; BMI: body mass index; CRP: C-reactive protein; F: factor; GGTP: gamma-glutamyltransferase; INR: international normalized ratio; LDL: low-density lipoprotein; PAI-1: plasminogen activator inhibitor-1; PLT: platelets; RBC: red blood cells; PT: prothrombotic time; SaO_2_: oxygen saturation; SVEF: single ventricular ejection fraction; TAFI: thrombin activatable fibrinolysis inhibitor; TC: total cholesterol; TG: triglycerides; tPA: tissue plasminogen activator; vWF: von Willebrand Factor; WBC: white blood cells.

**Table 3:** Baseline thromboembolic events profile of Fontan patients.

Thromboembolic Event	Fontan Patients, *n* (%)	Time from Procedure (Years)	Fenestration, *n* (%)	SV Type Left, *n* (%)	SVT, *n* (%)
PE	12 (13.2)	19.3 ± 4.9 [17.0–20.0]	6 (50.0)	8 (66.7)	3 (25.0)
IS/TIA	4 (4.4)	14.0 ± 5.2 [10.0–18.0]	3 (75.0)	4 (100.0)	2 (50.0)
FCT	6 (6.6)	18.2 ± 4.1 (16.0–20.0)	2 (33.3)	4 (66.7)	3 (50.0)
DVT	1 (1.1)	-	1 (100.0)	1 (100.0)	1 (100.0)
PST	1 (1.1)	-	1 (100.0)	1 (100.0)	1 (100.0)
All events	24 (26.4)	17.8 ± 5.2 [16.0–19.5]	12 (57.1)	15 (71.4)	9 (37.5)

Values are displayed as mean ± standard deviation, number (percentage), or median (interquartile range). DVT: deep venous thrombosis; FCT: Fontan circuit thrombus; IS: ischemic stroke; PE: pulmonary embolism; PST: peripheral systemic thrombosis SV: systemic ventricle; SVT: supraventricular tachycardia; TIA: transient ischemic attack.

**Table 4:** Thromboembolic events profile of Fontan patients during follow-up.

No	Time from Procedure (Years)	TE Type	History of Previous TE Event	SV Type	Fenestration	Antithrombotic Therapy	CHA_2_DS_2_-VASc (Points)	SVT (*n*)
1	22	PE	-	undefined	yes	Aspirin	0	1
2	14	PE	-	L	no	Aspirin	0	0
3	23	PE + DVT	-	R	no	Aspirin	2	1
4	15	PE	FCT	R	no	VKA	0	1
5	33	DVT	PE	L	no	NOAC	2	0
6	25	FCT + DVT	-	L	no	-	0	0
7	23	PE	PE + IS	L	no	NOAC	3	0

Values are displayed as mean ± standard deviation. DTV: deep vein thrombosis; FCT: Fontan circuit thrombus; IS: ischemic stroke; L: left; NOAC: novel oral anticoagulants; PE: pulmonary embolism; R: right; SV: systemic ventricle; SVT: supraventricular tachycardia; TE: thromboembolic; VKA: vitamin K antagonist.

## Data Availability

The data presented in this study are available on request from the corresponding author.
